# Polymorphisms in nucleotide excision repair genes and risk of primary prostate cancer in Chinese Han populations

**DOI:** 10.18632/oncotarget.13848

**Published:** 2016-12-10

**Authors:** Mengyun Wang, Qiaoxin Li, Chengyuan Gu, Yao Zhu, Yajun Yang, Jiucun Wang, Li Jin, Jing He, Dingwei Ye, Qingyi Wei

**Affiliations:** ^1^ Cancer Institute, Collaborative Innovation Center for Cancer Medicine, Fudan University Shanghai Cancer Center, Shanghai, China; ^2^ Department of Pathology, First Affiliated Hospital, Xinjiang Medical University, Urumqi, China; ^3^ Department of Urology, Fudan University Shanghai Cancer Center, Shanghai, China; ^4^ Ministry of Education Key Laboratory of Contemporary Anthropology, State Key Laboratory of Genetic Engineering, School of life Sciences, Fudan University, Shanghai, China; ^5^ Fudan-Taizhou Institute of Health Sciences, Taizhou, Jiangsu, China; ^6^ Department of Hepatobiliary Oncology, State Key Laboratory of Oncology in South China, Sun Yat-Sen University Cancer Center, Guangzhou, China; ^7^ Duke Cancer Institute, Duke University Medical Center, Durham, NC, USA; ^8^ Department of Oncology, Shanghai Medical College, Fudan University, Shanghai, China

**Keywords:** case-control study, prostate cancer, genetic susceptibility, nucleotide excision repair, polymorphism

## Abstract

Genetic variants of nucleotide excision repair (NER) genes have been extensively investigated for their roles in the development of prostate cancer (PCa); however, the published results have been inconsistent. In a hospital-based case-control study of 1,004 PCa cases and 1,055 cancer-free controls, we genotyped eight potentially functional single nucleotide polymorphisms (SNPs) of NER genes (i.e., *XPC*, rs2228001 T>G and rs1870134 G>C; *XPD*, rs13181 T>G and rs238406 G>T; *XPG*, rs1047768 T>C, rs751402 C>T, and rs17655 G>C; and *XPF*, rs2276464 G>C) and assessed their associations with risk of PCa by using logistic regression analysis. Among these eight SNPs investigated, only *XPC* rs1870134 CG/CC variant genotypes were associated with a decreased risk of prostate cancer under a dominant genetic model (adjusted odds ratio [OR] = 0.77, 95% confidence interval [CI] = 0.64–1.91, *P* = 0.003). Phenotype-genotype analysis also suggested that the *XPC* rs1870134 CG/CC variant genotypes were associated with significantly decreased expression levels of *XPC* mRNA in a mix population of different ethnicities. These findings suggested that *XPC* SNPs may contribute to risk of PCa in Eastern Chinese men.

## INTRODUCTION

Nucleotide excision repair (NER) is one of the most versatile, well-established DNA repair mechanisms in maintaining genomic stability and integrity [1]. NER is mainly responsible for repair of bulky, helix-distorting DNA damages, such as those caused by ultraviolet radiation, mutagenic chemicals, or chemotherapeutic drugs. Briefly, the repair process includes excision and removal of damaged nucleotides, synthesis of a short complementary sequence and substitution of the previously damaged DNA strand for the final restoration [1]. At least eight core genes (i.e., *ERCC1*, *XPA*, *XPB/ERCC3*, *XPC*, *XPD/ERCC2*, *XPE*, *XPF/ERCC4*, and *XPG/ERCC5*) derived from the xeroderma pigmentosm (XP) complementation group, together with other two genes (i.e., *CSA /ERCC8* and *CSB/ERCC6*) encoding proteins linked to Cockayne's syndrome, are involved in the pathway [2, 3], among which the *XP* genes were extensively investigated in the development of cancer, both *in vivo* and *in vitro*. These indicate that reduced DNA repair capacity phenotype may result in genomic instability and carcinogenesis by affecting repair proficiency and that genes involved in the NER pathway are likely to be involved in cancer susceptibility [1, 4–8].

While PCa is one of the most common human malignancies, ranking the second leading cause of cancer-related deaths among men in the US [9, 10], the incidence of PCa in developing countries had been increasing rapidly in the past decades [9, 11]. Although multiple factors are observed to be associated with PCa risk, little is known about the causes of the disease. The established environmental risk factors include age [12], ethnicity or geographic location [13–15], family history [16–18], physical inactivity, obesity and the increased intake of fat [19]. The exponential increase in PCa risk associated with aging may reflect the accumulation of DNA damage resulting from a series of biological aging processes, including increased oxidative stress, frequent inflammation, accumulated exposure to environmental carcinogens, or an age-related decreasing DNA damage-repair response [20–22]. In addition to environmental risk factors, genetic variation may also contribute to PCa susceptibility. For example, in the first genome-wide association studies (GWAS) in Han Chinese, it was found that two new risk-associated loci for PCa on chromosomes 9q31.2 (rs817826, *P* = 5.45 × 10^−14^) and 19q13.4 (rs103294, *P* = 5.34 × 10^−16^) in 4,484 PCa cases and 8,934 controls, in addition to confirming several associations reported in other ethnic groups [23]. These findings in the GWAS study improve our understanding of susceptibility to prostate cancer and promote further functional studies. Given that the NER mechanism is important in removal of oxidative DNA damage or DNA adducts in the genome [24], it is biologically plausible to speculate that germline variation in the NER genes may affect the capacity of their encoded DNA repair enzymes to effectively remove DNA adducts or lesions, subsequently leading to PCa risk.

Previous pre-GWAS studies have investigated associations between genetic variants in NER genes and PCa risk, but the results were inconsistent. For example, Mirecka et al. investigated 15 SNPs in seven XP genes (*XPA-XPG*) in a case-control study of 720 PCa patients and 1,121 cancer-free controls, and they found an increased risk of PCa was associated with the *XPD* SNP, rs1799793 (Asp312Asn) AG and AA genotypes [25]. In another study conducted by Hooker S et al., the homozygous variant genotype of *XPG/ERCC5* -72C/T promoter polymorphism was found to be associated with a significantly decreased risk of PCa [26]. More recently, Chen et al. performed a meta-analysis to evaluate three SNPs and their associations with risk of PCa and found no significant association between the *XPC* 939A/C polymorphism and PCa risk [27]. The results have been inconsistent due in part to population stratification, small sample size, inadequate statistical methods as well as limited study power to detect modest associations. Meanwhile, previous studies did not include gene-environment interaction analysis, and most of the SNPs selected were tagSNPs that may have no biological function. In the present study, we selected eight potentially functional SNPs in four NER genes (e.g., *XPC*, *XPD*, *XPF*, and *XPG*) to examine their associations with risk of PCa in a relatively large hospital-based case-control study of 1,004 PCa patients and 1,055 cancer-free controls of an Eastern Chinese Han population. Of these eight SNPs we selected, only rs2228001 and rs1318 have been studied in a previous Chinese study, in which the rs13181 G allele was found to be associated with a marginally increased risk of prostate cancer [28]. Other SNPs were not included in previously published pre-GWAS studies, nor in GWAS studies of Chinese populations or other populations. In the present study, therefore, we tested the hypothesis that the risk of PCa is associated with putatively functional SNPs in the NER genes, and we also assessed their interactions with environmental factors.

## RESULTS

### The *XPC* allele and genotype distributions and their associations with PCa risk

Among all of the eight SNPs under investigation, only *XPC* rs1870134 was associated with PCa risk, as summarized in Table 1. Briefly, a significant difference in genotype distribution between cases and controls was observed with more rs1870134 heterozygotes in control subjects than in case subjects (*P* = 0.015), and rs1870134 was associated with a decreased risk of prostate cancer, when the number of variant C alleles increased (*P*_trend_ = 0.007). A significantly decreased PCa risk associated with rs1870134 G>C was observed in both additive [adjusted OR = 0.82 (0.71-0.94), *P* = 0.0049] and dominant [adjusted OR = 0.77 (0.64-0.91), *P* = 0.003] genetic models.

**Table 1:** Logistic regression analysis of associations between NER variant genotypes and PCa risk in Eastern Chinese man

VariablesGenotypes	Cases(N=1004)	Controls(N=1055)	*P*	Crude OR(95% CI)	*P*^b^	Adjusted OR(95% CI)	*P*^c^
*XPC* rs1870134 (HWE: 0.424)							
GG	588 (58.6)	551 (52.2)	0.015^d^	1.00		1.00	
CG	356 (35.5)	430 (40.8)		0.78 (0.65-0.93)	0.006	**0.77 (0.64-0.93)**	**0.005**
CC	60 (6.0)	74 (7.0)		0.76 (0.53-1.09)	0.135	0.75 (0.52-1.07)	0.111
CG/CC	416 (41.4)	504 (47.8)		**0.77 (0.65-0.92)**	**0.004**	**0.77 (0.64-0.91)**	**0.003**
*XPC* rs2228001 (HWE: 0.379)							
TT	414 (41.2)	435 (41.2)	0.685^d^	1.00		1.00	
GT	459 (45.7)	495 (46.9)		0.97 (0.81-1.17)	0.783	0.98 (0.82-1.18)	0.850
GG	131 (13.1)	125 (11.9)		1.10 (0.83-1.46)	0.499	1.13 (0.85-1.49)	0.412
GT/GG	590 (58.8)	620 (58.8)		1.00 (0.84-1.19)	1.00	1.01 (0.85-1.21)	0.906
*XPD* rs13181 (HWE: 0.069)							
TT	845 (84.2)	907 (86.0)	0.259^d^	1.00		1.00	
GT	153 (15.2)	138 (13.0)		1.19 (0.93-1.53)	0.170	1.19 (0.93-1.52)	0.177
GG	6 (0.6)	10 (1.0)		0.64 (0.23-1.78)	0.396	0.64 (0.23-1.76)	0.382
GT/GG	159 (15.8)	148 (14.0)		1.15 (0.91-1.47)	0.250	1.15 (0.90-1.47)	0.262
*XPD* rs238406 (HWE: 0.240)							
GG	310 (30.9)	358 (33.9)	0.228^d^	1.00		1.00	
GT	480 (47.8)	497 (47.1)		1.12 (0.92-1.36)	0.279	1.13 (0.92-1.37)	0.244
TT	214 (21.3)	200 (19.0)		1.24 (0.97-1.58)	0.091	1.24 (0.97-1.59)	0.088
GT/TT	694 (69.1)	697 (66.1)		1.15 (0.96-1.38)	0.139	1.16 (0.96-1.39)	0.122
*XPG* rs17655 (HWE: 0.919)							
GG	231 (23.0)	272 (25.8)	0.342^d^	1.00		1.00	
CG	523 (52.1)	529 (50.1)		1.16 (0.94-1.44)	0.163	1.17 (0.94-1.45)	0.151
CC	250 (25.0)	254 (24.1)		1.16 (0.91-1.48)	0.244	1.17 (0.91-1.50)	0.222
CG/CC	773 (77.0)	783 (74.2)		1.16 (0.95-1.42)	0.144	1.17 (0.96-1.43)	0.131
*XPG* rs751402 (HWE: 0.834)							
CC	442 (44.0)	477 (45.2)	0.825^d^	1.00		1.00	
CT	458 (45.6)	467 (44.3)		1.06 (0.88-1.27)	0.543	1.05 (0.88-1.27)	0.577
TT	104 (10.4)	111 (10.5)		1.01 (0.75-1.36)	0.942	1.00 (0.75-1.35)	0.979
CT/TT	562 (56.0)	578 (54.8)		1.05 (0.88-1.25)	0.587	1.04 (0.88-1.24)	0.629
*XPG* rs1047768 (HWE: 0.461)							
TT	491 (48.9)	534 (50.6)	0.740^d^	1.00		1.00	
CT	433 (43.1)	440 (41.7)		1.07 (0.89-1.28)	0.461	1.07 (0.90-1.29)	0.447
CC	80 (8.0)	81 (7.7)		1.07 (0.77-1.50)	0.673	1.07 (0.77-1.49)	0.688
CT/CC	513 (51.1)	521 (49.4)		1.07 (0.90-1.27)	0.438	1.07 (0.90-1.28)	0.429
*XPF* rs2276464 (HWE: 0.153)							
GG	643 (64.0)	650 (61.6)	0.508^d^	1.00		1.00	
CG	328 (32.7)	366 (34.7)		0.91 (0.75-1.09)	0.295	0.90 (0.75-1.09)	0.284
CC	33 (3.3)	39 (3.7)		0.86 (0.53-1.38)	0.521	0.85 (0.53-1.37)	0.507
CG/CC	361 (36.0)	405 (38.4)		0.90 (0.75-1.08)	0.254	0.90 (0.75-1.08)	0.243

^b^ Unadjusted for age, smoking, and BMI status in logistic regress models.

^c^ Adjusted for age, smoking, and BMI status in logistic regress models.

^d^ For additive genetic models.

OR, odds ratio; CI, confidence interval; BMI, body mass index

The results were in bold, if the 95% CI excluded 1 or *P*<0.05.

### Stratified analysis of PCa risk associated with *XPC* SNPs

The multivariate logistic regression analyses with a dominant genetic model indicated that *XPC* rs1870134 CG/CC was associated with an decreased risk of PCa, particularly in subgroups of age > 65 [adjusted OR = 0.78 (0.63-0.97), *P* = 0.023], body mass index (BMI) ≤ 24kg/m^2^ [adjusted OR = 0.70 (0.56-0.86), *P* = 0.0007], ever smokers [adjusted OR = 0.75 (0.60-0.94), *P* = 0.012], and Gleason score ≤ 7(3+4) [adjusted OR = 0.72 (0.56-0.93), *P* = 0.012], compared with the homozygous wild-type genotype. However, further homogeneity tests indicated that there was no difference in risk estimates between subgroups for any of the strata, as shown in Table 2, suggesting that there were no evidence for any interactions between the SNPs and other covariates.

**Table 2:** Stratification analysis for associations between *XPC* variants and PCa risk by dominant genetic model in all subjects of Eastern Chinese man

Variables	rs1870134 (cases/controls)		CrudeOR (95%CI)	*P*	AdjustedOR (95%CI)^a^	P^a^	*P^hom^*
	CG/CC	GG					
Age,yr (median)							
<=65	137/178	199/191	**0.74 (0.55-1.00)**	**0.047**	**0.73 (0.54-0.98)**	**0.037**	0.725
>65	282/332	394/366	**0.79 (0.64-0.98)**	**0.029**	**0.78 (0.63-0.97)**	**0.023**	
BMI, kg/m2							
<=24	317/327	446/319	**0.69 (0.56-0.85)**	**0.0007**	**0.70 (0.56-0.86)**	**0.0007**	0.176
>24	102/183	147/238	0.90 (0.66-1.24)	0.526	0.90 (0.66-1.24)	0.521	
Smoking status							
Never	169/195	237/218	0.80 (0.61-1.05)	0.108	0.78 (0.59-1.04)	0.435	0.768
Ever	250/315	356/339	**0.76 (0.61-0.94)**	**0.014**	**0.75 (0.60-0.94)**	**0.012**	
Gleason score^b^							
<=7(3+4)	126/510	190/557	**0.72 (0.56-0.94)**	**0.013**	**0.72 (0.56-0.93)**	**0.012**	0.384
>=7(4+3)	262/510	342/557	0.84 (0.68-1.02)	0.082	0.83 (0.68-1.02)	0.069	

BMI, body mass index;

^a^ Obtained in logistic dominant models with adjustment for age, smoking status and BMI;

^b^According to the current WHO recommendations;

*P*^hom^=Homogeneiy test;

The results were in bold, if *P*<0.05.

### Correlation analysis of NER SNPs with mRNA expression

**Figure 1:**
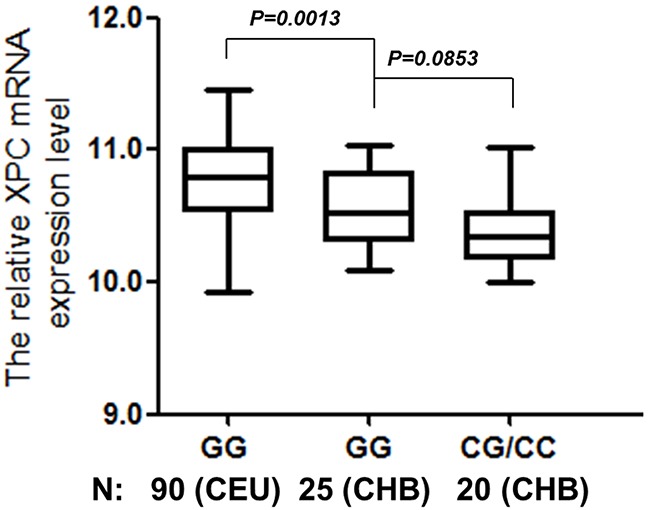
Correlation between *XPC* rs1870134 genotype and *XPC* mRNA expression for different populations (CEU and CHB)

**Figure 2:**
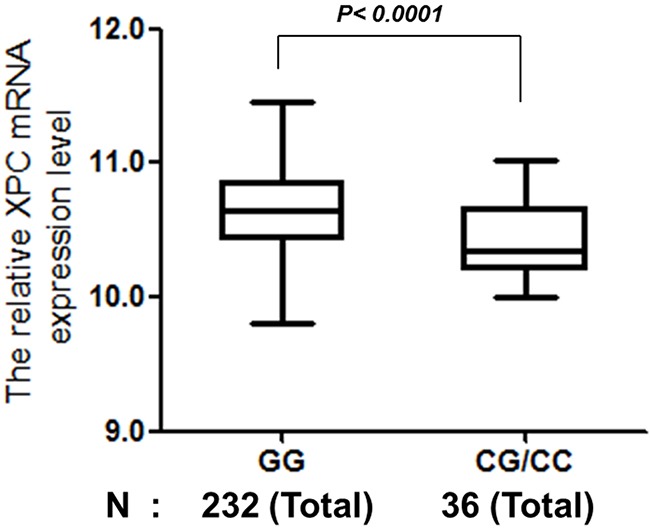
Correlation between *XPC* rs1870134 genotype and *XPC* mRNA expression for total populations

In the present study, we use public databases to evaluate the potential role of special SNPs in the gene expression. Briefly, the publically available databases for gene expression of the established lymphoblastoid cell lines were derived from 270 people with different ethnicities. The homozygous genotype was associated with higher *XPC* mRNA expression levels only in the Utah residents with ancestry from northern and western Europe (CEU) population (*P* = 0.0013), but not in Chinese Han in Beijing (CHB) population (Figure 1). A borderline significant difference in *XPC* mRNA expression levels was observed in Chinese participants carrying the rs1870134 GG genotype, compared with those carrying CG/CC genotypes (*P* = 0.0853), which is likely due to the small sample size (N = 45), and the overall genotype-phenotype correlation was present for all ethnic groups (N = 270, *P* < 0.0001, and R = 0.248) (Figure 2).

## DISCUSSION

The etiology of a complex disease like PCa usually involves multiple factors, such as multiple genes in multiple biological pathways in addition to environmental factors. Studies have identified that individuals with a decreased NER capacity are at an increased risk of cancer. Given the possibility that genetic variation in NER genes may contribute to variation in the NER capacity, the present study investigated the associations between eight potentially functional SNPs of the NER genes and PCa risk. The major finding was a significant association between rs1870134 G>C variant genotypes and a decreased risk of PCa under a dominant genetic model, and the phenotype-genotype analysis suggested that the *XPC* rs1870134 CG/CC variant genotypes were also associated with significantly decreased expression of *XPC* mRNA in all ethnic populations of the available datasets. The strengths of the present study include a relatively large sample size from a single institution and the analysis of gene-gene and gene-environmental interactions.

A number of association studies on the roles of NER polymorphisms in the etiology of PCa have been published in the last decades; however, inconclusive findings for the most frequently analyzed polymorphisms are evident among those published studies, even in some recently reported meta-analyses. Most of the reported meta-analyses for NER polymorphisms agreed that the NER polymorphisms were differentially associated with PCa risk by ethnicity. For *XPD*, the Asp312Asn (rs1799793) polymorphism was consistently shown to be associated with an increased risk of PCa in Asian populations in both additive and recessive genetic models [29–33] but not in Caucasian or African populations. For example, a protective association was observed between Asp312Asn (rs1799793) genotypes and PCa risk in Caucasian populations in one meta-analysis [31] but not in other three meta-analyses [29, 30, 33]. Likewise, an association was observed between Asp312Asn (rs1799793) genotypes and PCa risk in African populations in three meta-analyses [31–33] but not in other two meta-analyses [29, 30]. Similar to the previously published studies, we did not find any association between *XPD* Lys751Gln (rs13181) genotypes and PCa in Chinese populations. For *XPC*, a meta-analysis for Lys939Gln (rs2228001) [34] of 62 studies including 25708 cases and 30432 controls confirmed an increased cancer risk associated with this polymorphism in the homozygous genetic model for Asian populations, but not for other ethnic groups [35]. Taken together, there are some possible reasons for the inconclusive findings among these meta-analyses. Firstly, the heterogeneity test indicated heterogeneous results from different ethnic groups, which may result from their different genetic background or the environment the subjects lived in. Secondly, most of the sample sizes of the eligible studies included in the meta-analysis were too small for epidemiologic risk assessment. Thirdly, different inclusion strategy used by the meta-analyses and the undefined criteria for participants may also contribute to the heterogeneity. To our knowledge, our current study comprised a much larger sample size than most of reported studies by far for the association studies between NER polymorphisms and PCa in Chinese populations from a single institution. We found a significant association between rs1870134 G>C variant genotypes of *XPC* and a decreased risk of prostate cancer under a dominant genetic model in an Eastern Chinese population for the first time. This SNP was not included in previously published GWAS studies.

*XPC* located at chromosome 3p25 encodes a protein kinase product of 940 amino acids, which is an indispensable component of NER and is required for the early steps of DNA repair, especially in damage recognition and initiation of NER. According to an online server (http://www.ncbi.nlm.nih.gov/projects/SNP), 92 missense coding-SNPs were reported through across the whole *XPC* gene. SNPs rs1870134 is in the first exon, which was predicted as a tagSNP linking to 10 other tagged variants (rs3729584, rs3731091, rs2290711, rs3731123, rs3739588, rs3729585, rs1982546, rs3731115, rs3731174, and rs2045446). Among these, SNPs rs1982546 and rs2045446 were located in first intron and predicted to be involved in potential transcription factor binding sites. It is also possible that rs1870134 is in linkage disequilibrium (LD) with the real causal SNP located in the coding region and affects the protein function at the post-translational level. Thus, the sequencing of this gene will be necessary to identify additional causal variants in the future.

PCa is one of the common hereditary human malignancies with a high incidence in Caucasian and African populations, but with a much lower frequency in Asian populations. Interestingly, according to an *in silico* analysis, a different distribution of rs1870134 genotypes among ethnic populations was observed; for example, rs1870134 heterozygous and variant homozygous genotypes were not observed in African ancestry in Southwest (ASW) and CEU, but in Asian populations (i.e., Han Chinese and Japanese) (HapMap Data Rel 27 Phase II+III). In addition, according to an online SNPexp server system (http://app3.titan.uio.no/biotools), significant differences on *XPC* mRNA expression levels among populations carrying the wild homozygous genotypes were observed (Student *t-*test, *P* = 0.0013), with a lower *XPC* mRNA expression level in the CHB population, compared with that of the CEU population. Additionally, for CHB populations, we found that the C allele appeared to be correlated with a lower level of mRNA expression, compared with that of the G allele, although the difference was not statistically significant, likely due to small sample size and a weak allelic effect. Therefore, it is reasonable to speculate that the rs1870134 CG/CC genotypes might be associated with a decreased PCa risk by reducing *XPC* mRNA expression levels, or the decreased *XPC* mRNA expression levels might be linked to the variants (i.e., rs1982546 and rs2045446) that are true functional.

Recently, SNP rs2228001 of the *XPC* gene has been extensively investigated for its association with carcinogenesis of some tumor types (i.e., cancers of the bladder, lung, and colorectum); however, the findings were inconclusive with PCa. With the respect of PCa, only five studies by far have focused on the rs2228001 variant and PCa risk, and only one study observed that rs2228001CC frequency was significantly lower in PCa cases in a case-controls study of 165 cases and 165 controls [36]. However, no similar findings were observed by other investigators, including our current larger case-control study of 1,004 cases and 1,055 controls. Although the findings for *XPC* rs2228001 were inconsistent among published studies, a recent large meta-analysis found that rs2228001 CC carriers may have an increased cancer risk of several cancer types, especially for cancers of the bladder, lung, colorectum, compared with GG/GC carriers, indicating that the effects of *XPC* SNPs on cancer risk are unlikely to be tissue-specific [34]. However, the effects of *XPC* SNPs on cancer risk have not been fully validated, and further functional studies should be performed to unravel the underlying mechanisms.

The combined analyses in the present study further confirmed that a single SNP may have some weaker effect on cancer risk. Additionally, we noticed that the combined effect of two *XPC* SNPs was more pronounced among subgroups of age ≤ 69, ever smoker, and Gleason score ≤7(3+4). These findings agreed with the hypothesis that genetic susceptibility contributes to the risk of developing PCa in those who had an early age onset. Although the interaction between smoking and *XPC* SNPs was not observed in the present study (Supplementary Table S1), we did find an obvious effect of the combined unfavorable genotypes on PCa risk, particularly among subgroups of ever smokers, suggesting that the effect of the tobacco smoke-related carcinogens may also depend on genetic factors.

In summary, the present study investigated the associations between eight selected potentially functional NER SNPs and PCa risk, in which some moderate significant findings were observed. However, several methodological issues and limitations of the present study should be noticed. Firstly, some findings in the stratified analyses may be a chance finding due to the limited observations in the subgroups. Secondly, our present study is a retrospective study; therefore, some environment factors such as alcohol drinking were missing due to the inadequate information. Therefore, additional large and prospective studies, with carefully collection of detailed characteristics of the patients, are warranted to further confirm our findings. Meanwhile, the gene-environment interaction assessment in the present study was relatively crude with smoking described as ever/never. Also, we did not have much detailed exposure data that prevented us from effectively assessing gene-environment interactions. Third, there is a lack of validation of the results by another independent study population. We focused on an Eastern Chinese population, and we cannot found a similar study population or similar datasets available from other investigators. Because all these disadvantages and the limited number of SNPs of interest in the study can contribute to the false positive findings, we did perform the false positive probability reports to minimize possible false-positive associations. Fourthly, only eight SNPs of four NER genes were investigated in the present study, which did not cover all core genes in the NER pathway. Finally, we were not able to measure the mRNA expression of *XPC* using the real-time PCR method, due to the lack of clinic tissues/samples. Therefore, future studies should include such tissues to validate our findings.

## MATERIALS AND METHODS

### Patients and controls

The study included 1,004 patients with newly diagnosed and histopathologically confirmed primary PCa and 1,055 cancer-free controls. All of the eligible cases and controls were recruited from Shanghai Cancer Center, Fudan University (FUSCC) and Taizhou longitudinal study (TZL), respectively, between January 2008 and January 2012. The entire document and inclusion criteria for participants were described previously [23]. Briefly, 1,004 PCa patients and 1,055 cancer-free controls were well matched by age (±5 years). Among the case subjects, there were more higher grade PCa than lower grade ones, of which 551 (54.4%) cases had prostate specific antigen (PSA) ≥ 20 ng/ml and 604 (59.7%) with Gleason score ≥ 7 (4+3). However, 87 (8.6%), 92 (9.1%), and 81 (8.0%) cases had missing information about serum PSA values, Gleason scores, and clinical staging status, respectively, due to insufficient documented records.

All study subjects signed a written informed consent when approached for participation of the study, and the research was approved by the Institutional Review Board of Fudan University Shanghai Cancer Center. And the methods were carried out in accordance with the approved guidelines.

### SNP selection and genotyping

Potentially functional SNPs in NER genes (e.g., *XPC*, *XPD*, *XPF*, and *XPG*) of interest were selected for genotyping in the present study according to the following criteria: (a) potentially functional significance predicted by SNPinfo online server (http://snpinfo.niehs.nih.gov/snpfunc.htm), (b) MAF reported in HapMap was ≥ 5% for Chinese subjects, (c) LD coefficient *r*^2^<0.8 between SNPs, and (d) not included in the reported GWASs. Only SNPs agreed with these criteria were finally selected. Additionally, SNPs associated with carcinogenesis of any tumor form were optimally considered in this study.

We used the SNPinfo online server (http://snpinfo.niehs.nih.gov/snpfunc.htm) and NCBI dbSNP database (http://www.ncbi.nlm.nih.gov/sites/entrez?db=Snp) to assess the potential function of the target SNPs and HaploView 4.2 software (http://www.broadinstitute.org/mpg/haploview) to calculate the linkage between SNPs. Ultimately; we selected eight SNPs of interest for further investigation. For the *XPC* gene, two tagging nsSNPs (none-synonymous SNP, e.g. rs2228001T>G and rs1870134G>C) were selected. For the *XPD* gene, two nsSNPs (rs13181T>G and rs238406 G>T) involving potential exonic splicing enhancer/silencer (ESE/ESS) loci were selected. For the *XPG* gene, two SNPs (rs1047768 T>C and rs751402 C>T) involving potential ESE/ESS loci and one nsSNP (rs17655 G>C) were selected. For the *XPF* gene, one SNP (rs2276464G>C) located in the 3′-untranslated region (3′ UTR) of *XPF*, involve in the miRNA binding site, was selected.

Subsequently, bioinformatics analysis was followed by using HaploView software 4.2 to estimate the haplotype block for CHB population (HapMap Data Rel 27 Phase II+III), and no LD was found between any of these SNPs described above. In the current study, DNA isolation was performed with the buffy-coat fraction of the blood samples donated by the subjects included in this study with the Qiagen Blood DNA Mini KIT (Qiagen Inc., Valencia,CA). Genotyping was performed using the TaqMan real-time PCR method with genotyping master mix and pre-designed primers and probes for each SNP purchased from ABI (Applied Biosystems, Foster City, CA), as described previously [23], and the results with >98% call rates and 98.5% or greater agreement for duplicated specimens were acceptable for further genotyping data analysis.

### Genotype and mRNA expression data of lymphoblastoid cell lines from HapMap database

To explore biological plausibility of the studied SNPs, we used publically available genotyping data from the HapMap phase 2 release 23 data set (http://www.sanger.Ac.uk/humgen/hapmap3) based on four ethnicity-specific populations (90 CEU for Utah residents from northern and western Europe; 45 CHB for unrelated Han Chinese in Beijing; 45 JPT for unrelated Japanese in Tokyo; 90 YRI for unrelated Yorba in Ibadan, Nigeria). The linear regression model-based trend test was performed to assess the correlation between genotypes of the select gene and its mRNA expression available online (http://app3.titan.uio.no/biotools/tool.php?app=snpexp).

### Statistical analysis

Deviation from Hardy-Weinberg Equilibrium (HWE) among controls for each SNP analysis was assessed by a goodness-of-fit χ^2^ test. Distributions of the alleles, genotypes and the categorical variables of interest between cases and controls were evaluated by Pearson's χ^2^ test. Traditional genetic models for association studies were conducted, including dominant, recessive, and additive models. To confirm individual effect of each SNP on PCa risk, crude ORs and 95% confidence intervals (CIs) were calculated using unconditional logistic regression models with adjustment for age, smoking status and BMI. Further stratified analyses were conducted by univariate and multivariate unconditional logistic regression methods on the best-fitting genetic models, evaluated by likelihood-ratio based estimates, to assess the associations. Finally, the homogeneity Q-test was used to identify any difference between the strata.

All statistical analyses were performed with SAS 9.1 statistical software (Cary, NC, USA). All tests were two-sided, and a *P* value of <0.05 was considered statistically significant.

## SUPPLEMENTARY MATERIALS FIGURES AND TABLES



## Abbreviations

SNPs: single nucleotide polymorphisms
PCa: prostate cancer
OR: odds ratio
CI: confidence interval
NER: nucleotide excision repair
HWE: Hardy-Weinberg equilibrium
