# Supplementary material for: Polymorphisms in nucleotide excision repair genes and risk of primary prostate cancer in Chinese Han populations
Source: Oncotarget. 2016 Dec 10;8(15):24362–71. doi: 10.18632/oncotarget.13848 (PMC5421853; doi:10.18632/oncotarget.13848)
Supplement: Supplementary file 1 [file oncotarget-08-24362-s001.pdf]

Polymorphisms in nucleotide excision repair genes and risk of primary prostate cancer in Chinese Han populations

SUPPLEMENTARY TABLE

Supplementary Table 1: Stratification analysis of significant SNPs by age, smoking status, and BMI

Variables	Age yr	Cases (n=1004)	Controls (n=1055)	Adjusted OR (95% CI)*	Interaction P^a
rs1870134 × age					0.653
GG	≤ 65	198 (19.7)	190 (18.0)	1.00	
GG	>65	390 (38.8)	361 (34.2)	1.02(0.80-1.30)	
CG/CC	≤65	137 (13.7)	178 (16.9)	0.74 (0.55-0.99)	
CG/CC	>65	279 (27.8)	326 (30.9)	0.80 (0.62-1.03)	
rs1870134 × smoking status					0.713
GG	Never	232 (23.1)	215 (20.4)	1.00	
GG	Ever	356 (35.5)	336 (31.9)	1.00 (0.79-1.27)	
CG/CC	Never	166 (16.5)	191 (18.1)	0.80 (0.60-1.05)	
CG/CC	Ever	250 (24.9)	313 (29.7)	0.75 (0.58-0.96)	
rs1870134 × BMI					0.092
GG	≤24	441 (43.9)	312 (29.6)	1.00	
GG	>24	147 (14.6)	239 (22.7)	0.44 (0.34-0.57)	
CG/CC	≤24	314 (31.3)	321 (30.4)	0.69 (0.56-0.86)	
CG/CC	>24	102 (10.2)	183 (17.4)	0.40 (0.30-0.53)	

*Data were calculated by unconditional logistic regression, adjusting for age, smoking status and BMI.
[a]*P* value for Factor 1 × Factor 2 presents a test for the interaction between Factor 1 and Factor 2. The results were in bold, if $P<0.05$.
